# Steroid Receptor Coregulators Can Modulate the Action of Progesterone Receptor during the Estrous Cycle in Cow Endometrium

**DOI:** 10.3390/ani11113217

**Published:** 2021-11-10

**Authors:** Robert Rekawiecki, Karolina Dobrzyn, Magdalena K. Kowalik

**Affiliations:** Institute of Animal Reproduction and Food Research of the Polish Academy of Sciences, Tuwima 10, 10-747 Olsztyn, Poland; k.dobrzyn@pan.olsztyn.pl (K.D.); m.kowalik@pan.olsztyn.pl (M.K.K.)

**Keywords:** progesterone receptor coregulators, P300, CREB, SRC-1, NCOR-2, endometrium, cow

## Abstract

**Simple Summary:**

Proper functioning of the endometrium is necessary for the implantation of the embryo after fertilization and its development throughout pregnancy. The key role in this process plays appropriate action of progesterone through the nuclear receptor isoforms. The action of the receptor is regulated by the attachment of receptor modulators called coregulators which include coactivators and corepressors. Their improper expression in humans causes a malfunction of progesterone receptors and leads to disorders of pregnancy. However, in farm animals, such disorders may be one of the reasons leading up to early embryonic lethality, which in cows reaches up to 40%. Obtained results indicate the important role of the studied coregulators in regulating progesterone activity in endometrial cells, especially during the preimplantation period. Therefore, they can be helpful in better understanding the regulation and expression of the coactivators and corepressors in cow endometrium during the estrous cycle and can contribute to reducing this problem. They can also be of significant practical importance, making for the increased efficiency of breeding these animals.

**Abstract:**

Nuclear receptor coregulators include coactivators and corepressors which associate with the progesterone receptor (PGR) during its activation. Fluctuations in the transcription levels of their respective genes and subsequent protein production as well as in related activities for histone acetyltransferase (HAT) and histone deacetylase (HDAC) can affect PGR function and thus change the action of progesterone (P4) in bovine endometrium during the estrous cycle. Endometrial tissue on days 2–5, 6–10, 11–16, and 17–20 of the estrous cycle was used for determination of the mRNA expression levels of coactivators P300, CREB, and SRC-1 along with corepressor NCOR-2 using Real-Time PCR, with protein levels by Western blot. Coregulators cellular localizations were assessed by immunohistochemistry whereas the activities of HAT and HDAC by using EIA. The highest levels of mRNA and proteins for all of the investigated coregulators, as well as the highest levels of activity for HAT and HDAC, were detected over days 2–16 of the estrous cycle. All of the tested coregulatory proteins were localized in the nuclei of endometrial cells. This research indicates the important role of coregulators of the PGR receptor in regulating P4 activity in endometrial cells, especially during the pre-implantation period.

## 1. Introduction

The endometrium lines the interior of the uterus; its structure and function change periodically throughout the estrous cycle. Proper functioning of the endometrium is necessary for the implantation of the embryo after fertilization and its development over the course of pregnancy. The hormone responsible for creating the necessary conditions is P4, which is synthesized primarily in CL and enters the uterus via counter-current transfer [1,2]. Because this compound is a steroid hormone, it works primarily through nuclear receptors (PGR), which are classified as transcription factors. By linking to the promoter of a given gene, the PGR receptor regulates that gene’s transcription. Based on their protein structures, two receptor isoforms can be distinguished: isoform A (PGRA) and isoform B (PGRB). The amino acid sequence of PGRB is longer than that of PGRA by approximately 164 amino acids in humans; in other species, this difference varies from 128 to 165 amino acids [3]. While the same gene encodes both isoforms, transcription for either PGRA or PGRB begins with two separate promoters [3]. Apart from their architecture, they also differ in their action. Progesterone mostly acts through the PGRB isoform, and PGRA acts as an inhibitor of PGRB’s action [4,5]. Both PGR isoforms have been localized in the endometrium and show variable expression throughout the estrous cycle, as detected in the human endometrium [6]. The final step in the activation of PGR is the attachment of receptor function modulators called coregulators. This group of proteins include coactivators—whose attachment activates transcription of a given gene—and corepressors, which have the opposite effect [7]. Coregulators are not specific to the PGR receptor; they operate throughout the group of steroid hormone receptors, whose actions they can also regulate, as with estradiol [8], androgen [9], or glucocorticoid receptors [10]. Coactivators show histone acetyltransferase activity (HAT): through the acetylation of histone proteins, they induce the transition from heterochromatin to euchromatin [11]. In contrast, corepressors show histone deacetylase (HDAC) activity—by deacetylation of histone proteins, they increase the amount of heterochromatin, thus limiting the transcription of various genes [12]. Their proper function in regulating transcription is important in both animals and humans, and any disruption in their functioning can lead to breast, ovarian, or endometrial cancers, in addition to various genetic diseases such as Rubenstein-Taybi syndrome [13,14]. Therefore, the obtained results can be a model for research on pathological conditions of the uterus in both cows and humans.

Our earlier studies have shown that the genes for the coactivator P300/CBP-associated factor (PCAF) and for the corepressor Nuclear Receptor Corepressor (NCOR1) undergo variable expression in cow endometrium throughout the estrous cycle [15]. Thus it is possible, that the changes in mRNA and protein levels of other coregulators and related HAT and HDAC activities may affect on the endometrial PGR function thus modulating the impact of P4 on target cells. Therefore, our aims in this study were to evaluate cellular distribution, measure the mRNA and protein levels of expanded group of coregulators containing: coactivators—histone acetyltransferase p300 (P300), cAMP response element-binding protein (CREB), and steroid receptor coactivator-1 (SRC-1)—as well as the corepressor nuclear receptor corepressor-2 (NCOR-2) in endometrial tissue during the estrous cycle in a cow. Another aim was to determine HAT and HDAC activity in the same tissue in the course of the cycle.

## 2. Materials and Methods

### 2.1. Tissue Collection

Endometrial slices from non-pregnant cows and mature heifers were collected from an industrial slaughterhouse and taken to the laboratory in liquid nitrogen. Tissues from the four stages of the estrous cycle (Days 2–5, 6–10, 11–16, and 17–20) were stored at −80 °C for future experiments. The estrous cycle stages were assessed based on the changes in morphology of the ovaries and uteri based on the guidelines described in [16,17], respectively. Endometrial sections were transported to the laboratory in ice-cold phosphate-buffered saline (PBS) and were used to isolate nuclear proteins as material to determine HAT and HDAC activity. However, as a material for immunohistochemistry Endometrial tissue were fixed with 4% paraformaldehyde in 0.1 M PBS (pH 7.4) for 24 h and next rinsed with distilled water, followed by dehydration using ethanol gradient and finally embedding in paraffin which were used to prepare microscopic slides for immunohistochemistry. The tissues frozen in liquid nitrogen were homogenized with a Retsch MM-2 vibratory mill (Retsch GmbH, Haan, Germany). The obtained tissue powder was divided into portions for the isolation of RNA and for protein level determination. Nuclear proteins were isolated by means of the Nuclear/Cytosol Fractionation Kit (Biovision, Milpitas, CA, USA) according to the manufacturer’s instructions, and stored at −80 °C for future research.

### 2.2. Progesterone Concentrations

The determination of the P4 level was performed by enzyme immunoassay (EIA) using a microplate reader (Multiscan EX, Labsystem, Helsinki, Finland) and absorbance was measured at 450 nm wavelength. Petroleum ether was used to extract the hormone from the endometrial tissue [18], and the recovery of this hormone averaged 90%. The final dilution of horseradish peroxidase-labeled P4 amounted to 1:40,000. Whereas P4 antiserum (IFP4) was applied in dilution 1:60,000 as was described by [19]. The standard curve was between 0.1–25 ng/mL, while the sensitivity of the method was 0.15 ng/mL. Hormone level was converted per gram of endometrial tissue. The control samples had intra- and inter-assay coefficients of variation accordingly on levels 6.2% and 7.7. The relationship between the amount of hormone added and measured (*n* = 5) was significant (r = 0.96).

### 2.3. Immunohistochemistry

Uterine sections 6 µm thick were cut from paraffin-embedded tissue blocks and mounted on Super Frost Plus microscope slides. Sections were deparaffinized with xylene and rehydrated in a series of ethanol dilutions (100%, 96%, 70%, H2O). Antigen retrieval was performed by sections cooking in 0.01 M sodium citrate with 0.05% Tween 20 (pH 6.0) 3× for 5 min each time. Blocking of endogenous peroxidase activity was performed with BLOXALL Endogenous Peroxidase reagent and Alkaline Phosphatase Blocking Solution (Vector Laboratories) for 15 min. After this time, the sections were incubated for 45 min with 2.5% normal horse serum (NHS) to block the antibody’s non-specific binding sites. The sections prepared in this way were incubated overnight at 4 °C with primary antibodies against the following proteins: P300 (dilution 1:70) (Cohesion Biosciences, London, UK, catalog CPA1389), CREB (dilution 1:400) (Cohesion Biosciences, London, UK, catalog CPA1273), SRC-1 (dilution 1:35) (Sigma, Poznan, Poland, catalog HPA070520), and corepressor NCOR–2 (dilution 1:50) (Sigma, Poznan, Poland, catalog HPA001928). To check for non-specific signals the negative control was used in which the antibodies were replaced with 2.5% of normal horse serum (NHS). Residues of antibodies were rinsed in TBS 3 × 10 min and next the ImmPRESS reagent (Vector Laboratories) was added and slides were incubated at 4 °C for 30 min. This reagent is a secondary antibody with a unique micropolymer chain consisting of densely packed, highly active peroxidase molecules. After this time slides were washed for 3 × 10 min in TBS. The visualization of the antigens was done using 3,3′-diaminobenzidine (DAB; Vector Laboratories, Burlingame, CA, USA). Finally, they were rinsed, counterstained with Mayer’s hematoxylin (1 min), dehydrated, and embedded in a mounting medium (DPX, POCh, Gliwice, Poland). Pictures were taken with a Zeiss Axio Imager Z1 microscope (Zeiss, Jena, Germany).

### 2.4. RNA Isolation and Reverse Transcription

The Universal RNA Purification Kit (EURx, Gdańsk, Poland) was used to isolate total RNA. The procedure was carried out in accordance with the manufacturer’s instructions based on the previously described method [20]. A NanoDrop 1000 spectrophotometer (Thermo Scientific, Wilmington, DE, USA) was used to measure the concentrations and check the purity of RNA which then was stored at −80 °C for further experiments. Obtained RNA was treated with DNase (1 µg) to get rid of residual genomic DNA. The reverse transcription process was performed using the TRANSCRIPTME cDNA Synthesis Kit (Blirt, Gdańsk, Poland) according to the manufacturer’s recommendations.

### 2.5. Real-Time PCR

The 7900 real-time PCR System (Applied Biosystems, Foster City, CA, USA), was used to perform real-time PCR analyzes. Reactions were carried out with the Power SYBR Green PCR Master Mix (Applied Biosystems, Foster City, CA, USA). Oligonucleotide primers for PCR and lengths of individual amplification products for coregulators: P300, CREB, SRC-1, and NCOR-2, including the TATA box-binding protein (TBP), applied as a housekeeping gene were presented in Table 1. The single reaction mixture (20 µL) consisted of 100 ng cDNA, 10 μL Master Mix (Applied Biosystems, Foster City, CA, USA), and 0.2 mM of each primer for the appropriate gene. The PCR reaction began with the initial denaturation step (10 min w 95 °C), and then 40 denaturation cycles (15 s in 95 °C) as well as annealing and extension (1 min at 60 °C). All PCR reactions (*n* = 5) were performed in duplicate.

Real-time PCR to determine PGRA and PGRB mRNA levels were performed using TaqMan probes. Table 1 shows the sequences of the primers and probes together with the sizes of the PCR amplification products for PGRA, PGRB, and the housekeeping gene TBP. The volume of the real-time PCR reaction mixture was 20 µL and it consisted of: 10 µL of Applied Biosystems TaqMan Fast Universal PCR Master Mix (Applied Biosystems, Foster City, CA, USA), 0.2 mM probe and primers for each of the tested genes as well as cDNA 100 ng. The PCR reaction was run under the following conditions: initial denaturation step (10 min at 95 °C), followed by 40 cycles of denaturation (15 s at 95 °C), and hybridization and extension (1 min at 60 °C.) All PCR amplifications were performed in duplicate.

### 2.6. Western Blot Analysis

Preparation of protein extracts consisted in homogenization of endometrial tissues in the radioimmunoprecipitation assay buffer (RIPA) with protease inhibitors (25 mM Tris-HCl, pH 7.6; 150 mM NaCl, 1% Triton X-100, 1% sodium deoxycholate, and 0.1% sodium dodecyl sulfate [SDS]). Proteins at 100 mg per well were electrophoresed on a 7.5% SDS-polyacrylamide gel (SDS-PAGE) precast Stain-Free gels (Biorad, Hercules, CA, USA). In the next step, the proteins were subjected to wet transfer from the gel to an Immobilon polyvinylidene fluoride (PVDF) membrane (Millipore, Billerica, MA, USA). A 5% non-fat dry milk in Tris-buffered saline and Tween 20 (TBST) buffer (100 mM Tris-HCl, 0.9% NaCl, and 0.05% Tween 20) was used to block the PVDF membrane. Next, the membranes were incubated overnight at 4 °C with the same antibodies as were used in the IHC analysis except the NCOR-2 antibody. We used the following antibodies: anti-P300 produced in rabbit (dilution 1:400), which recognizes the P300 protein with a molecular mass of approximately 264 kDa; anti-CREB produced in rabbit (dilution 1:130), with a molecular mass of approximately 265 kDa; SRC-1 (dilution 1:200) with a molecular mass of approximately 152 kDa. For detection of NCOR-2 protein we used ani- NCOR-2 antibody, with a molecular mass of approximately 274 kDa (dilution 1:400) (OriGene Technology, Rockville, MD, USA, catalog TA330553). After the end of incubation, the membranes were washed three times for 10 min with TBST buffer. After this step, the membranes were treated with secondary antibodies horseradish peroxidase (HRP) conjugated anti-rabbit IgG (Sigma, Poznan, Poland, catalog A6154) at a dilution of 1:50,000. The last step was a reaction with the Clarity Western ECL Blotting chromogenic substrate (Biorad, Hercules, CA, USA) to detect the studied protein. The product of the proteolytic reaction was light which was produced proportionally to the amount of HRP-labeled antibodies, the signal was visualized with a ChemiDoc Imaging System (Biorad, Hercules, CA, USA). The level of each of the tested proteins was normalized to the total level of loaded protein in each gel well using ImageLab analysis software (Biorad, Hercules, CA, USA). The Stain-Free method did not require any additional use of a housekeeping protein antibodies [21].

### 2.7. HAT and HDAC Activity

The activity of the HAT and HDAC enzymes were determined with using ready-made kits such as the HAT Activity Colorimetric Assay Kit (Biovision, Milpitas, CA, USA) and the HDAC Activity Colorimetric Kit (Biovision, Milpitas, CA, USA). The experiments were carried out in accordance with the manufacturer’s procedures. Previously isolated extracts of nuclear proteins were used as a material for the research.

### 2.8. Data Analysis

The graphs presented in the publication show the mean ± SEM values of hormone concentrations, real-time PCR, and Western blot. One-way analysis of variance (ANOVA) was used for the statistical analysis, followed by the Tukey test. Quantitative data from real-time PCR experiments were analyzed using the real-time PCR Miner algorithm [22] and then normalized to TBP. Western blot measurements were normalized to the total amount of proteins separated on the acrylamide gels using Stain-Free technology and protein chemiluminescent detection. Graph Pad Prism 8.0 software (GraphPad Software, Inc., San Diego, CA, USA) was used for statistical analyses of the obtained results.

## 3. Results

### 3.1. Immunohistochemistry

**Figure 1:**
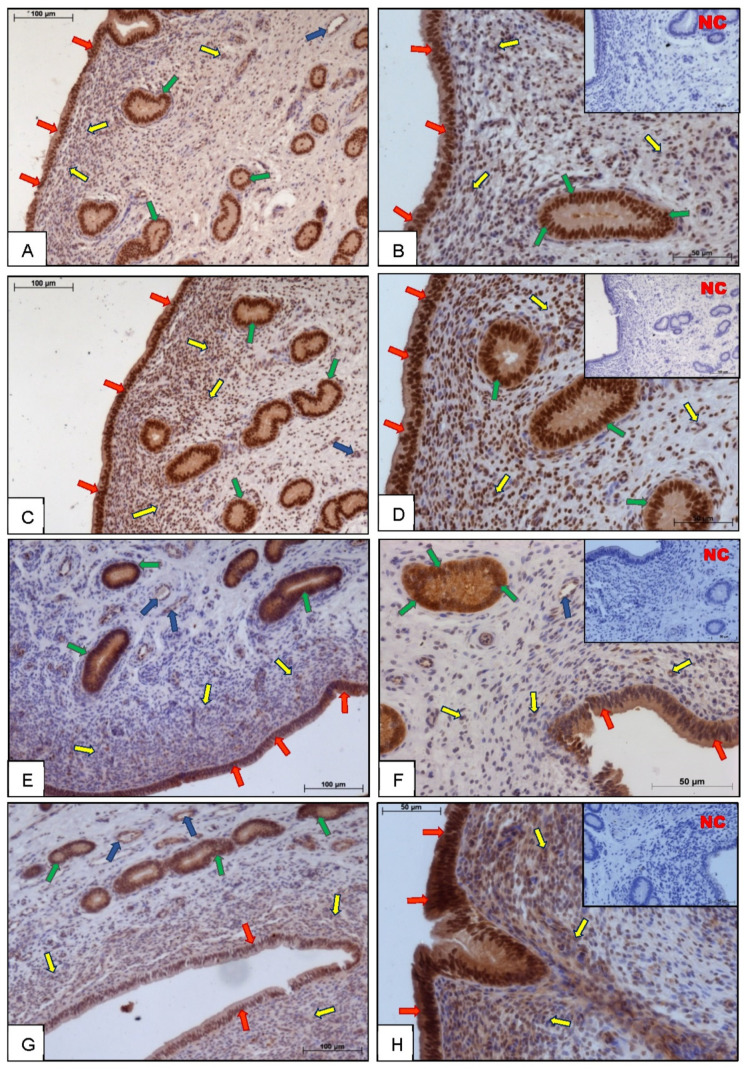
The cellular localization of coactivators: histone acetyltransferase p300 (P300) (**A**,**B**), cAMP response element-binding protein (CREB) (**C**,**D**), steroid receptor coactivator-1 (SRC-1) (**E**,**F**), and corepressor nuclear receptor corepressor-2 (NCOR-2) (**G**,**H**) in bovine endometrium on days 6–10 of the estrous cycle. Negative control (NC) was performed without primary antibodies (smaller box inside right pictures). Red arrows indicate luminal epithelium; blue arrows indicate endothelial cells of blood vessels, yellow arrows indicate stromal cells and green arrows indicate glandular cells. Scale bars: 100 μm (picture on the left) and 50 μm (picture on the right).

Immunostaining for P300 (Figure 1A,B), CREB (Figure 1C,D), SRC-1 (Figure 1E,F), and NCOR-2 (Figure 1G,H) was performed on endometrial slices on days 6–10 of the estrous cycle, showing their nuclear localization in epithelial cells and in the glandular, stromal, and endothelial cells of blood vessels in cow endometrium. Negative control sections for all investigated proteins were consistently free of stain.

### 3.2. Progesterone Concentrations

**Figure 2:**
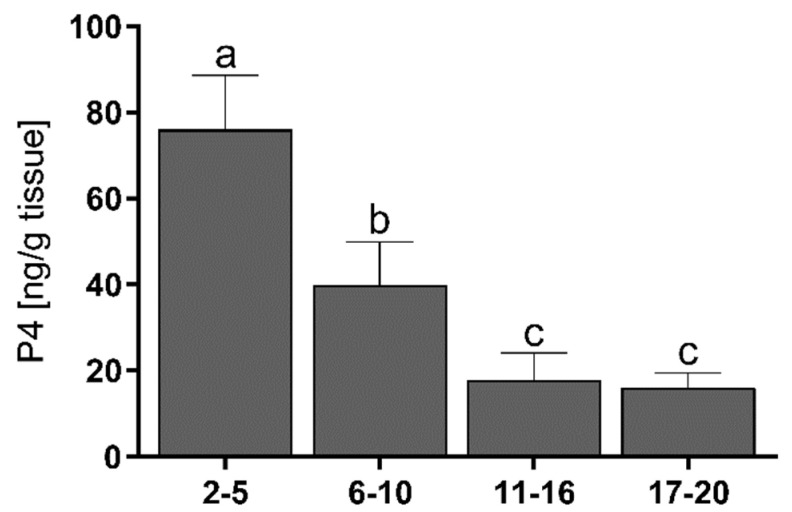
The mean (±SEM) progesterone (P4) concentrations in bovine endometrium collected on days 2–5, 6–10, 11–16, and 17–20 (*n* = 5 per stage) of the estrous cycle. Statistical significance was assessed using one-way ANOVA followed by the Tukey test. Different letters a, b and c represent statistical siginificance (*p* < 0.05).

The endometrial concentration of P4 was the highest on days 2–5 of the estrous cycle, followed by a decrease on days 6–10 (*p* < 0.001) to its lowest level on days 11–20 (*p* < 0.05) (Figure 2).

There were positive correlations between the P4 concentrations and the mRNA levels of SRC-1 (*p* < 0.0001) and NCOR-2 (*p* < 0.001). Correlations were also found between P4 and the protein levels of CBP (*p* < 0.0001), SRC-1 (*p* < 0.0001), and NCOR-2 (*p* < 0.0001) (Table 2).

### 3.3. mRNA Levels of Coregulators, PGRA, and PGRB Isoforms in the Endometrium

**Figure 3:**
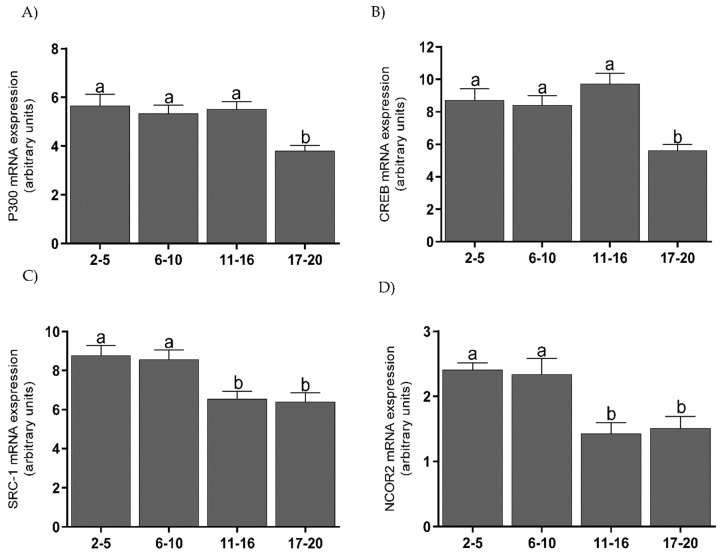
The mean (±SEM) mRNA levels for P300 (**A**), CREB (**B**), SRC–1 (**C**), and NCOR–2 (**D**) in bovine endometrium, collected on days 2–5, 6–10, 11–16, and 17–20 (*n* = 5 per stage) of the estrous cycle. Statistical significance was assessed using one-way ANOVA followed by the Tukey test. Different letters a and b represent statistical significance (*p* < 0.05).

**Figure 4:**
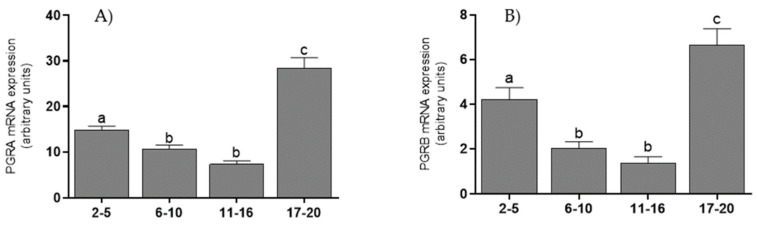
The mean (±SEM) mRNA levels for PGRA (**A**) and PGRB (**B**) in bovine endometrium, collected on days 2–5, 6–10, 11–16, and 17–20 (*n* = 5 per stage) of the estrous cycle. Statistical significance was assessed using one-way ANOVA followed by the Tukey test. Different letters a, b and c represent statistical significance (*p* < 0.05).

Endometrial tissue showed the highest levels of mRNA for P300 (Figure 3A) and CREB (Figure 3B) on days 2–16, which were decreased on days 17–20 (*p* < 0.001). For mRNA transcripts of SRC-1 (Figure 3C) and NCOR-2 (Figure 3D), levels were highest on days 2–10 and lower during days 11–16 (*p* < 0.05); they remained at this level until the end of the cycle. mRNA levels for PGRA were high on days 2–5, lower on days 6–16 (*p* < 0.01), and increased on days 17–20 (*p* < 0.05) (Figure 4A). mRNA levels for PGRB were also high on days 2–5, lower on days 6–16 (*p* < 0.01), and increased on days 17–20 (*p* < 0.001) (Figure 4B).

There was a negative correlation between PGRA mRNA levels and NCOR-2 mRNA levels (*p* < 0.05). Negative correlations were also found between PGRB mRNA levels and mRNA levels for CBP (*p* < 0.05), SRC-1 (*p* < 0.05), and NCOR-2 (*p* < 0.05) (Table 2).

### 3.4. The Protein Levels of Coregulators in the Endometrium

**Figure 5:**
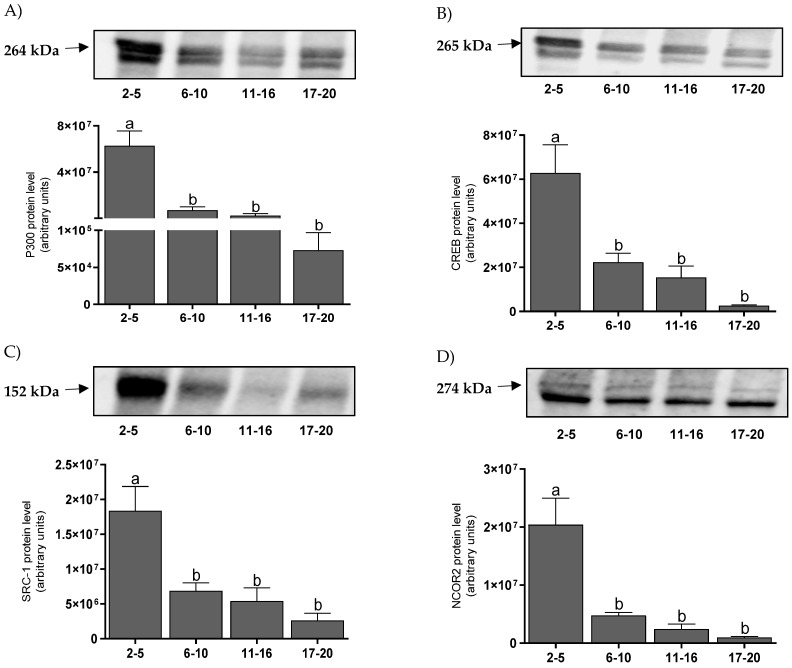
The mean (±SEM) protein levels of P300 (**A**), CREB (**B**), SRC–1 (**C**), and NCOR–2 (**D**) in bovine endometrium, collected on days 2–5, 6–10, 11–16, and 17–20 (*n* = 5 per stage) of the estrous cycle. Statistical significance was assessed using one-way ANOVA followed by the Tukey test. Different letters a and b represent statistical significance (*p* < 0.05).

The protein levels of coactivators in the endometrium were highest on days 2–5 for P300 (Figure 5A), CREB (Figure 5B), and SRC-1 (Figure 5C). All of the protein levels decreased on days 6–10 (*p* < 0.001) and remained at the same level until the end of the cycle. The protein level for the NCOR-2 corepressor was highest on days 2–5 and fell on days 6–10 (*p* < 0.01) and remained at the same low level until the end of the cycle (Figure 5D). There were positive correlations between mRNA and protein levels for P300 (*p* < 0.01) (Table 2).

### 3.5. Histone Acetyltransferase and Histone Deacetylase Activities

**Figure 6:**
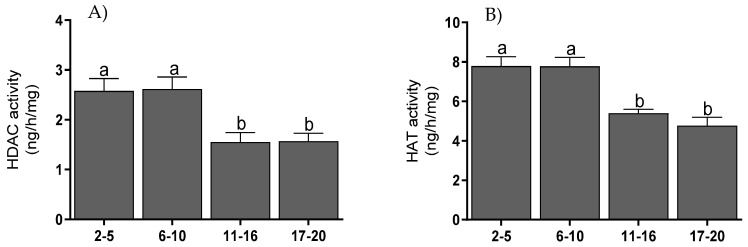
HAT (**A**) and HDAC (**B**) activity in bovine endometrium collected on days 2–5, 6–10, 11–16, and 17–20 (*n* = 5 per stage) of the estrous cycle. Statistical significance was assessed using one-way ANOVA followed by the Tukey test. Different letters a and b represent statistical significance (*p* < 0.05).

The activity of HAT (Figure 6A) in endometrial tissue was highest on days 2–10 (*p* < 0.001) and was decreased on days 11–16 (*p* < 0.01) and 17–20 (*p* < 0.001). The activity of HDAC (Figure 6B) in the endometrium was highest on days 2–10 and decreased on days 11–16 (*p* < 0.05) and on days 17–20 (*p* < 0.01).

There were positive correlations between HAT activity and protein levels for CBP (*p* < 0.0001) and NCOR-2 (*p* < 0.05). Correlations were also found between HDAC activities and protein levels of CBP (*p* < 0.001), SRC-1 (*p* < 0.05), and NCOR-2 (*p* < 0.05). Both HAT and HDAC activities were also mutually positively correlated (*p* < 001) (Table 2).

## 4. Discussion

Immunohistochemical analysis for P300, CREB, SRC-1, and NCOR-2 showed intense staining of cell nuclei in epithelial, stromal, as well as in endothelial cells of blood vessels in cow endometrium. The obtained results confirm the localization of the studied coregulatory proteins in the nuclei of uterine cells, as in bovine luteal cells [23]. A localization pattern similar to coregulators has also been demonstrated for PGR receptors [24,25]. This suggests a possible interaction between coregulators and PGR and supports the action of P4 in endometrial cells.

The mRNA levels of SRC-1 and NCOR-2 and those of the CREB, SRC-1, and NCOR-2 proteins correlated with the P4 level. Only the P300 protein level showed no statistical differences during the cycle and no correlation with the hormone level. PGRA and PGRB mRNA levels were highest at the beginning and end of the estrous cycle, as found previously in published studies on the protein levels of these receptors in the bovine endometrium [26]. Increased expression of these isoforms is associated with intensive preparation of the uterus for possible pregnancy [27]. The obtained results also show lower expression of PGRB mRNA compared to PGRA mRNA. PGRA acts as a potent inhibitor of PGRB’s action, thus reducing the effect of P4 on target cells [5,28]. Therefore, levels of PGRA higher than those of PGRB may be one of the mechanisms regulating the action of P4, as was shown in our previous research on cow CL [29].

In the endometrium, most of the studied coregulators showed a correlation between mRNA or protein levels and the level of P4. Similar relationships were found in our previous research on the P300/CBP-associated factor (PCAF) coactivator and Nuclear Receptor Corepressor 1 (NCOR1) [15]. Additionally, some of the coactivators showed a negative correlation between their mRNA levels and the mRNA level of the PGRA and PGRB isoforms. Therefore, in both the endometrium and the CL [23], we can also suppose that the expression of coregulators may depend on the level of P4 but not on the mRNA expression level of the PGRA and PGRB isoforms.

Although coregulators do not attach to the PGR receptor individually, they form a complex of proteins that supports activation. SRC-1, P300, and CREB are part of such a protein complex that binds to the PGR receptor dimer after connecting to the hormone response element in the promoter of the activated gene [30]. Thus, the similar levels of mRNA and coactivator proteins obtained in our research on endometrium and the bovine CL [23] may indicate an equal participation of different coregulatory proteins in PGR receptor activation.

The highest levels of P300 and CREB mRNAs were detected on days 2–16, whereas the highest levels of SRC-1 mRNA occurred on days 2–10. For each of these, the mRNA levels decreased in the final days of the cycle. Progesterone synthesized in CL enters the uterus by countercurrent transfer from lymphatic and venous vessels to the uterine arterial system; it prepares the uterus for implantation of an embryo [1,2]. In the absence of fertilization, the secretion of prostaglandin F2α (PGF2α) increases; as a consequence of its action, P4 secretion in CL decreases [31]. This intensifies apoptotic processes in endometrial cells and decreases the levels of mRNA transcripts and protein isoforms of the PGR receptor [26,32]. However, a pre-ovulatory increase in the estradiol (E2) level raises endometrial PGR protein levels in the final days of the cycle [33]. The highest levels of mRNA transcripts and proteins of the investigated coactivators in our research were found only at the beginning of the cycle. Therefore, PGR coactivators may support the action of PGR isoforms in creating appropriate conditions for embryo implantation. However, in the last stage of the cycle, their activity is minimal and is aimed at maintaining the activity of the PGR receptor before the next ovulation.

Coregulators are involved in the regulation of not only nuclear PGR but also of other nuclear steroid hormone receptors as estrogen receptors [34]. High levels of estradiol receptor protein were found during and several days after the estrous cycle, and also at the end of the cycle [33]. A pre-ovulatory increase in E2 levels stimulates the proliferation of endometrial cells in the process of endometrial remodeling [35]. Therefore, coregulators may support the activity of both P4 and E2 receptors in the endometrium of cows during the estrous cycle.

Corepressors show the opposite effect of coactivators by inhibiting the transcription of the nuclear receptor to which they bind [12]. Our results show a positive correlation between the levels of mRNA and protein of the NCOR-2 corepressor and the levels of mRNA and proteins of the three tested coactivators. Both coactivators and corepressors bind to the nuclear receptor within the LBD (ligand-binding domain). Part of this domain, called the nuclear receptor box (NR box), is responsible for the attachment of coactivators; it consists of three leucine amino acids and two non-specific amino acids forming the LXXLL sequence [36,37]. Corepressors have additional amino acids that externally surround this motif to form (I/L)XX(I/V)I [38]. The failure to attach a ligand or the action of a receptor antagonist may result in a conformational change in the coregulator binding site and the formation of the corresponding space for the binding of corepressors. Reference [39] also indicate that a mutation in the coactivator binding site in the human thyroid receptor (TRb) inhibits the attachment of both coactivators and corepressors. Therefore, correlations between the mRNA and corepressor protein levels and the levels of mRNA and coactivator proteins may indicate that their binding to the receptor may occur competitively.

The levels of mRNA and protein for P300 show a negative correlation: the protein level for this coactivator decreased earlier than the mRNA level. No correlation was found between mRNA and protein levels for the other investigated coactivators and corepressor. This negative correlation may suggest the existence of molecular mechanisms for translational control. One such mechanism may be negative feedback between the protein level (gene product) and mRNA synthesis from that gene. High protein levels inhibit self-gene transcription, as shown with the c-myc oncogene [40]. Differences in the relationship between mRNA levels and the corresponding protein may also depend on the many complex and diverse regulatory mechanisms involved in the translation of mRNA into protein. However, these mechanisms are not yet understood sufficiently to enable direct calculations of protein concentrations from mRNA levels [41].

Coactivators are related to the action of the enzyme histone acetyltransferase (HAT), which causes the relaxation of the chromatin structure by acetylation of histone proteins and facilitates the access of transcription machinery to a gene’s DNA. On the other hand, corepressors are associated with histone deacetylase (HDAC), which has the opposite effect of HAT [42]. The obtained results indicate the highest levels of HAT and HDAC activity on days 2–10 of the cycle. In human endometrium, increased HAT activity at the early stage of proliferation may be associated with the increased transcriptional activity of genes involved in endometrial regeneration [43,44]. The subsequent decrease in acetylation in the secretory phase was associated with the regression of the corpus luteum and changes in endometrial function related to the absence of pregnancy [43]. Our research shows a correlation between the levels of HAT and HDAC activity. Maintaining cellular homeostasis requires a balance between HAT and HDAC activity [45]. Any disturbance of this balance may be associated with overexpression or inhibited expression of genes critical for proper cellular functioning [46]. Therefore, the correlation between the activity profiles of HAT and HDAC in our research may indicate an element of this balance between the activities of various genes necessary for cellular functioning.

## 5. Conclusions

The mRNA and protein levels of the studied coregulators show changes dependent on the level of P4, indicating the role of this hormone in the regulation of their expression. The similar expression profiles of the coactivators and corepressor may indicate their competitive binding to the PGR receptor. On the other hand, the action of HAT and HDAC, with similar levels of activity, may suggest a mechanism for maintaining homeostasis: the activation and inhibition of the expression of genes crucial for proper cellular functioning.

## Figures and Tables

**Table 1:** Forward, reverse primers sequences used in real Time PCR. Every primer set was designed according to accession number in the Nucleotide NCBI database.

Gen Name	Primers	GenBank Accession Number	Amplicon Length
P300	Forward: CCATGAGCAACATGAGTGCTAGT Reverse: CATTGTCACTCATCAGTGGGTTTT	XM_027540695.1	129
CBP	Forward: TGAAGTGAAGGTCGAAGCTAAAGA Reverse: GTACAGAGCTTCCAGGGTTGACAT	XM_024984694.1	147
SRC-1	Forward: CCCAGGCAGACGCTAAACAG Reverse: TCAAGATAGCTTGCCGATTTTG	XM_028514416.1	114
NCOR-2	Forward: AGCCCTCGAGGCAAAAGC Reverse: CATGCGGAGAGGCCTTGA	XM_024977670.1	177
TBP	Forward: CAGAGAGCTCCGGGATCGT Reverse: ACACCATCTTCCCAGAACTGAATAT	NM_001075742	194
PGRB	Custom Plus TaqMan RNA, Assay ID: AJY9X9P		-
PGRA	Forward: GGCAATTGGTTTGAGGCAAA Reverse: TCTTGGGTAACTGTGCAGCAA Probe: TTGTCCCTAGCTCACAGCGTTTCTATCAGC	AJ557823.1	196
TBP	Forward: CAGAGAGCTCCGGGATCGT Reverse: ACACCATCTTCCCAGAACTGAATAT Probe: AATCCCAAGCGTTTTGCTGCTGTAATCA	NM_001075742	194

**Table 2:** Coefficients of correlation for the progesterone (P4), progesterone isoform A (PGRA) mRNA progesterone isoform B (PGRB) mRNA, histone acetyltransferase (HAT) activity and histone deacetylase (HDAC) activity and mRNA and protein levels of coactivators of histone acetyltransferase p300 (P300), cAMP response element-binding protein (CREB), steroid receptor coactivator-1 (SRC-1), and nuclear receptor corepressor-2 (NCOR Abbreviation: ns, not significantly different.-2) and correlation for HAT and HDAC activities in cow endometrium during the estrous cycle.

	P300	CBP	SRC-1	NCOR-2
P4-mRNA	ns	ns	r = 0.70 *p* < 0.0001	r = 0.61 *p* < 0.001
P4-protein	ns	r = 0.69 *p* < 0.0001	r = 0.83 *p* < 0.0001	r = 0.78 *p* < 0.0001
mRNA-protein	r = −0.57 *p* < 0.01	ns	ns	ns
HAT-mRNA	ns	ns	ns	ns
HAT-protein	ns	r = 0.72 *p* < 0.0001	ns	r = 0.56 *p* < 0.05
HDAC-mRNA	ns	ns	ns	ns
HDAC-protein	ns	r = 0.60 *p* < 0.001	r = 0.49 *p* < 0.05	r = 0.51 *p* < 0.05
HAT-HDAC	r = 0.58 *p* < 0.001			
PGRA mRNA-mRNA	ns	ns	ns	r = −0.54 *p* < 0.05
PGRB mRNA-mRNA	ns	r = −0.50 *p* < 0.05	r = −0.58 *p* < 0.05	r = −0.65 *p* < 0.05

## Data Availability

All the data presented in this study are included in the article.
